# Preparation of cell-permeable Cre recombinase by expressed protein ligation

**DOI:** 10.1186/s12896-015-0126-z

**Published:** 2015-02-19

**Authors:** Soo Kyung Lyu, Hyockman Kwon

**Affiliations:** Department of Bioscience and Biotechnology and Protein Research Center for Bio-Industry, Hankuk University of Foreign Studies, Yongin, 449-791 Republic of Korea

**Keywords:** Expressed protein ligation, Cre recombinase, Polyarginine, Cell-penetrating peptides, Intracellular delivery

## Abstract

**Background:**

Protein transduction is safer than viral vector-mediated transduction for the delivery of a therapeutic protein into a cell. Fusion proteins with an arginine-rich cell-penetrating peptide have been produced in *E. coli*, but the low solubility of the fusion protein expressed in *E. coli* impedes the large-scale production of fusion proteins from *E. coli*.

**Results:**

Expressed protein ligation is a semisynthetic method to ligate a bacterially expressed protein with a chemically synthesized peptide. In this study, we developed expressed protein ligation-based techniques to conjugate synthetic polyarginine peptides to Cre recombinase. The conjugation efficiency of this technique was higher than 80%. Using this method, we prepared semisynthetic Cre with poly-L-arginine (ssCre-R9), poly-D-arginine (ssCre-dR9) and biotin (ssCre-dR9-biotin). We found that ssCre-R9 was delivered to the cell to a comparable level or more efficiently compared with Cre-R11 and TAT-Cre expressed as recombinant fusion proteins in *E. coli*. We also found that the poly-D-arginine cell-penetrating peptide was more effective than the poly-L-arginine cell-penetrating peptide for the delivery of Cre into cell. We visualized the cell transduced with ssCre-dR9-biotin using avidin-FITC.

**Conclusions:**

Collectively, the results demonstrate that expressed protein ligation is an excellent technique for the production of cell-permeable Cre recombinase with polyarginine cell-penetrating peptides. In addition, this approach will extend the use of cell-permeable proteins to more sophisticated applications, such as cell imaging.

**Electronic supplementary material:**

The online version of this article (doi:10.1186/s12896-015-0126-z) contains supplementary material, which is available to authorized users.

## Background

Protein delivery is regarded as a safer and useful alternative to gene delivery for the induction of genome editing and alterations in the gene expression profile of cells. Gene delivery through the transfection of plasmid DNAs or viral transductions inevitably imposes potential complications to gene alteration and is subject to safety problems. In contrast, there is a slight possibility that a genome would be altered by proteins delivered directly into cells. Furthermore, proteins become degraded and are eventually removed from cells. This property makes protein delivery more suitable for cell therapy applications in which a transient, rather than a sustained, action of transcription factors is required.

Due to the impermeability of proteins through a nonpolar cell membrane, protein delivery typically requires a protein delivery vector. Cell-penetrating peptides (CPPs) have emerged as the most promising protein delivery vector and are distinct from liposomes and other encapsulation technologies. CPPs are defined as short peptides (10–30 amino acids) that facilitate the cellular uptake of large macromolecules with a low membrane permeability [1]. Since the discovery that Tat48–60 enables the HIV-1 Tat protein to penetrate cells and activates HIV-1-specific target genes [2-4], several CPPs, including penetratin and transportan, have been reported. CPPs are either arginine-rich, amphipathic, or hydrophobic [5]. Recently, a cell-type-specific CPP, Xentry, is found from the X-protein of the hepatitis B virus [6]. The arginine-rich CPPs are short basic peptides highly enriched with arginine and lysine residues. The guanidinium groups of arginines play a central role in the cell penetrating activity of the arginine-rich CPPs, and in fact, homooligomers of arginine (7–12 arginines) are significantly more effective than native arginine-rich CPPs, such as Tat_48–60_ [7,8].

The different characteristics in the peptide sequences of CPPs suggest that their translocation mechanisms are not identical. Most importantly, the arginine-rich CPPs are less toxic than the amphipathic CPPs, and the arginine-rich CPPs do not cause membrane leakage at low micromolar concentrations where the arginine-rich CPPs are highly effective for cellular uptake [9-11]. Considering their low toxicity and high efficiency, the arginine-rich CPPs have been preferentially selected as protein delivery vectors. For example, the polyarginine fusion proteins of Oct4, Sox2, Klf4, and c-Myc have been delivered into somatic cells to reprogram them into iPSCs [12,13], and TAT-Pdx1, TAT-Ngn3, and TAT-MafA have been used to stimulate pancreatic β-cell differentiation [14-16]. Moreover, R7-CARM1 has been delivered to human mesenchymal stem cells to modulate their differentiation potential [17].

The arginine-rich CPP proteins have been produced in *E. coli* as recombinant fusion proteins. However, the fusion of the arginine-rich CPPs to proteins markedly decreases the solubility of the proteins, and when these are over-expressed in *E. coli*, they generally form the inclusion bodies. To obtain the soluble and active form of the proteins, the inclusion bodies have to be solubilized by denaturation agents, such as urea, and then the proteins have to be refolded [12,14,16,18]. Alternatively, the temperature used to induce protein expression has to be lowered to maintain the solubility of the arginine-rich CPP proteins when expressed in *E. coli* [15,17]. However, either method severely decreases the efficiency of the recovery of the active proteins, which impedes the large-scale production of the arginine-rich CPP proteins from *E. coli*.

**Figure 1:**
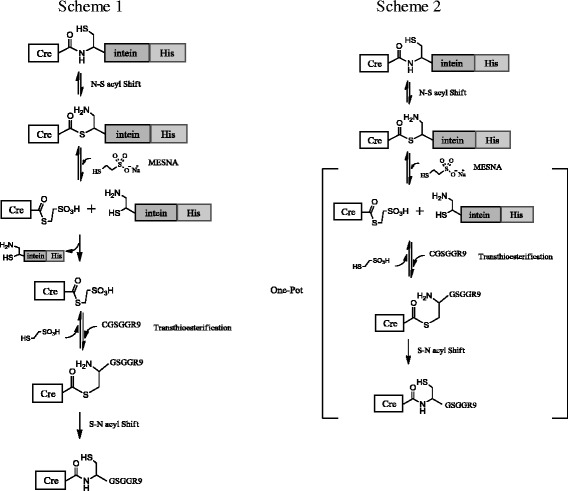
**General scheme for expressed protein ligation.** In scheme 1, the Cre-thioester is isolated from the intein-His for further reaction with the thiol group of an N-terminal cysteine of the peptides. In scheme 2, the thiolysis by MESNA, the transthioesterification reaction, and the S-N acyl Shift proceed in one-pot.

Expressed protein ligation (EPL) is a semisynthetic technique that conjugates a synthetic peptide containing an N-terminal cysteine to a C-terminal thioester through a native chemical ligation reaction (Figure 1). A C-terminal thioester of a protein is generated by the thiolysis of a recombinant intein fusion protein expressed in *E. coli*. The orthogonal reactivity, the production of a native peptide bond, and the high yield in an aqueous buffer at neutral pH and low temperature make EPL a powerful ligation method that introduces a synthetic peptide into recombinant proteins. EPL has been used to incorporate unnatural amino acids, fluorophores, and post-translational modifications to proteins [19]. In this study, we employed EPL to conjugate a synthetic polyarginine CPP polypeptide to a recombinant protein expressed in a soluble form in *E. coli*. The major advantage of this EPL-based technique over the preparation of recombinant polyarginine CPP fusion proteins from *E. coli* is that the EPL-based technique is not subject to the solubility problem as long as protein can be produced as soluble form in *E. coli*. In addition, the EPL-based technique allows the preparation of cell-permeable proteins with novel arginine-rich CPPs composed of unnatural amino acids, guanidinium-rich peptoids, or biotin.

The applicability of the EPL-based technique for the preparation of arginine-rich CPP proteins was previously tested using the enhanced green fluorescent protein (EGFP) as a model protein [20]. The flow cytometry study showed that EGFP conjugated with arginine 12-mer (R12) prepared by the EPL-based technique was delivered to cells with an efficiency comparable to that obtained with EGFP-R12 expressed as a fusion protein in *E. coli*. However, the EGFP-R12 molecules taken up by cells appeared to accumulate at the cell surface or be entrapped in endosomes, and it was uncertain whether these were delivered to the cytoplasm. The bio-activity of EGFP-12R in the cells was not examined in the study.

The Cre recombinase from bacteriophage P1 is a DNA sequence-specific recombinase that catalyzes the recombination of DNA between two loxP sites. Cre has been widely used to generate a tissue-specific or inducible knockout in transgenic mice or conditional gene expression in cells. Cre carries the nuclear localization sequence (NLS)-like elements that direct the efficient entry of Cre protein into the nucleus of mammalian cells [21]. In addition, cells harboring a loxP-STOP-loxP reporter gene, such as β-galactosidase or EGFP, allow us to monitor Cre-mediated recombination by Cre protein [22,23]. In this study, we prepared a cell-permeable Cre protein through the conjugation of synthetic polyarginine CPPs to recombinant Cre expressed in *E. coli* using EPL. The semisynthetic Cre-R9 (ssCre-R9) prepared by EPL had the similar enzymatic activity as Cre. We found that ssCre-R9 efficiently led to recombination events in living cells. It was previously reported that *d-isomer* polyarginine CPPs are more efficient than *l-isomer* polyarginine CPPs for the delivery of fluorescently labeled peptides [7]. Biotin (vitamin H or B-7) is a water-soluble vitamin, and the strong biotin-avidin interaction allows imaging of the cells that uptake a biotin-containing protein. Because EPL allows the incorporation of *d-isomer* amino acids and peptide mimetics into a protein, we prepared Cre proteins with the *d-isomer* of R9 (ssCre-dR9) and biotin (ssCre-dR9-biotin) by EPL and explored whether these elaborations in the resulting CPP enhance the effectiveness of the intracellular delivery or allow cell imaging.

## Results and discussion

**Figure 2:**
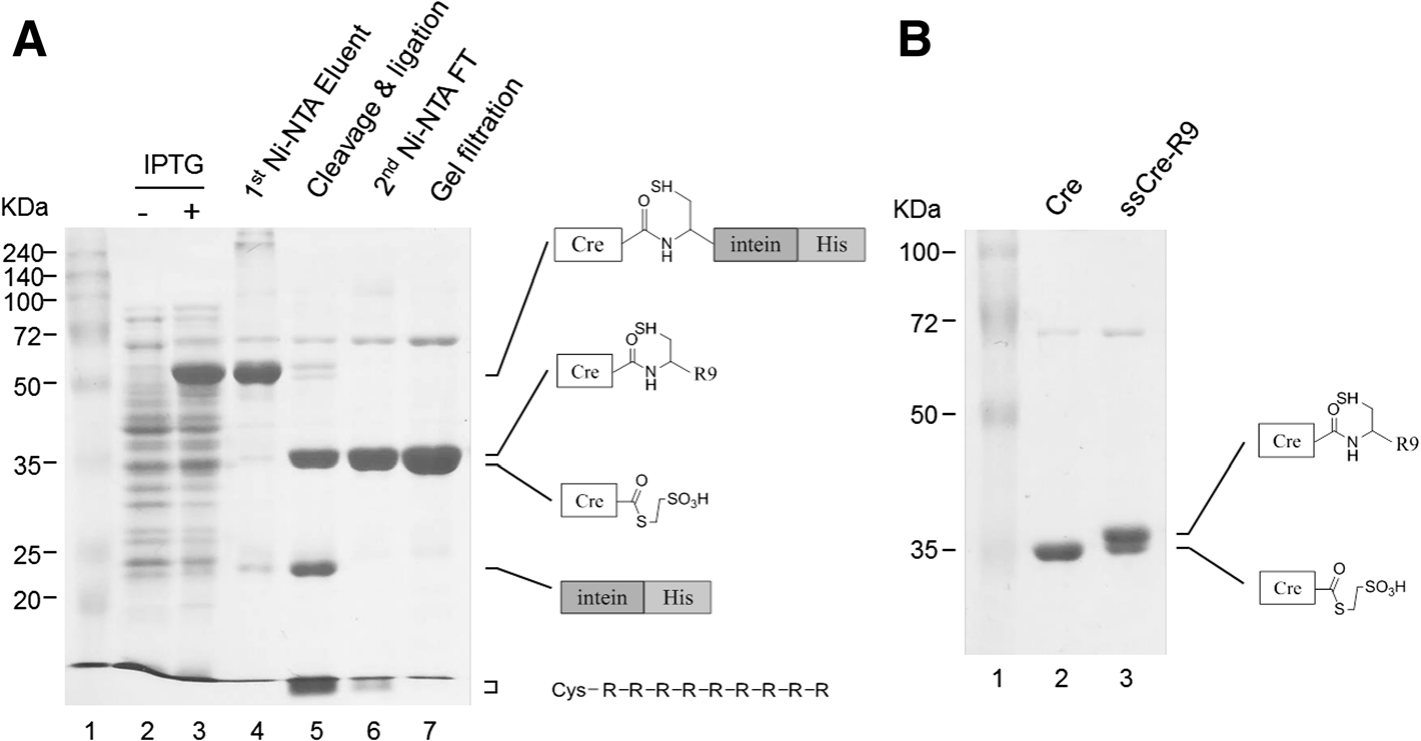
**Semi-synthesis of Cre-R9 by EPL. (A)** Expression of Cre-intein-His was induced by the addition of IPTG (lane 3). Cre-intein-His was purified by Ni-NTA chromatography (lane 4). After dialysis, Cre-intein-His was cleaved by MESNA and ligated with CGSGG-R9 peptide at 4°C for 24 h (lane 5). Cre-R9 was isolated from intein-His and CGSGG-R9 peptide by Ni-NTA chromatography (lane 6) and gel filtration (lane 7). Lane 1, size marker. The 12% SDS-PAGE gel was stained with Coomassie Brilliant Blue R-250. **(B)** The 12.5% SDS-PAGE gel was run for a longer running time, and it separated Cre-R9 from Cre-thioester as the distinct bands (lane 3). Cre was prepared by the DTT-mediated cleavage of Cre-intein-His (lane 2). Lane 1, size marker. The gel was stained with Coomassie Brilliant Blue R–250. The ratio of Cre-R9:Cre-thioester, which was estimated from the integrated peak areas of densitometry analysis of lane 3, was 84:16.

The Cre-intein-His protein was expressed in *E. coli* and purified in its native form by Ni^2+^-agarose column chromatography. We generated a Cre-thioester by intein-mediated cleavage using MESNA and then ligated the resultant Cre-thioester with CGSGG-R9 or CGSGG-dR9 by native chemical ligation. To minimize any potential adverse effect of MESNA on the activity of Cre, we first performed the intein-mediated cleavage reaction with 50 mM MESNA at 4°C for 24 h. Under this condition, more than 95% of the Cre-intein-His proteins were cleaved into Cre and the intein-His (lane 4 and lane 5 of Figure 2A). Second, we explored the direct conversion of the Cre-intein-His protein into the ligation product of Cre-R9 through a two-step one-pot reaction (Scheme 2 of Figure 1). EPL proceeds through the following three reactions; the intein-mediated cleavage reaction by thiol reagents, the transthioesterification reaction between the thiol group of an N-terminal cysteine of the peptides and the Cre-thioester, and the spontaneous intramolecular S → N acyl shift that generates an amide bond at the ligation junction. The first two reactions are reversible, but the last reaction is irreversible. The irreversibility of the last amide-forming reaction allows the two-step one-pot reaction, in which the intein-mediated cleavage and the native chemical ligation proceed in one pot, resulting in a high yield of the final ligation product. We conducted the two-step one-pot EPL reaction with 2 mg/ml Cre-intein-His and a 10-fold excess of CGSGG-R9 in 50 mM MESNA, 50 mM NaH_2_PO_4_, and 250 mM NaCl, pH 8.0 at 4°C for 24 h. The intein-His proteins, MESNA, and CGSGG-R9 were then removed from the final ligation product by Ni^2+^-agarose column chromatography and subsequent gel filtration chromatography (Figure 2A). Cre-R9 migrates slowly compared to Cre and forms the distinct up-shifted band on SDS-PAGE (Figure 2B). Conjugation efficiency was calculated from integrated peak areas of densitometry analysis of the Coomassie-stained SDS-PAGE gels. Typically, 84% of Cre was conjugated into Cre-R9, and the final yield of Cre-R9 was more than 2 mg from 20 mg of the Cre-intein-His protein. We named the final Cre-R9 product produced by EPL as the semisynthetic Cre-R9 (ssCre-R9).

**Figure 3:**
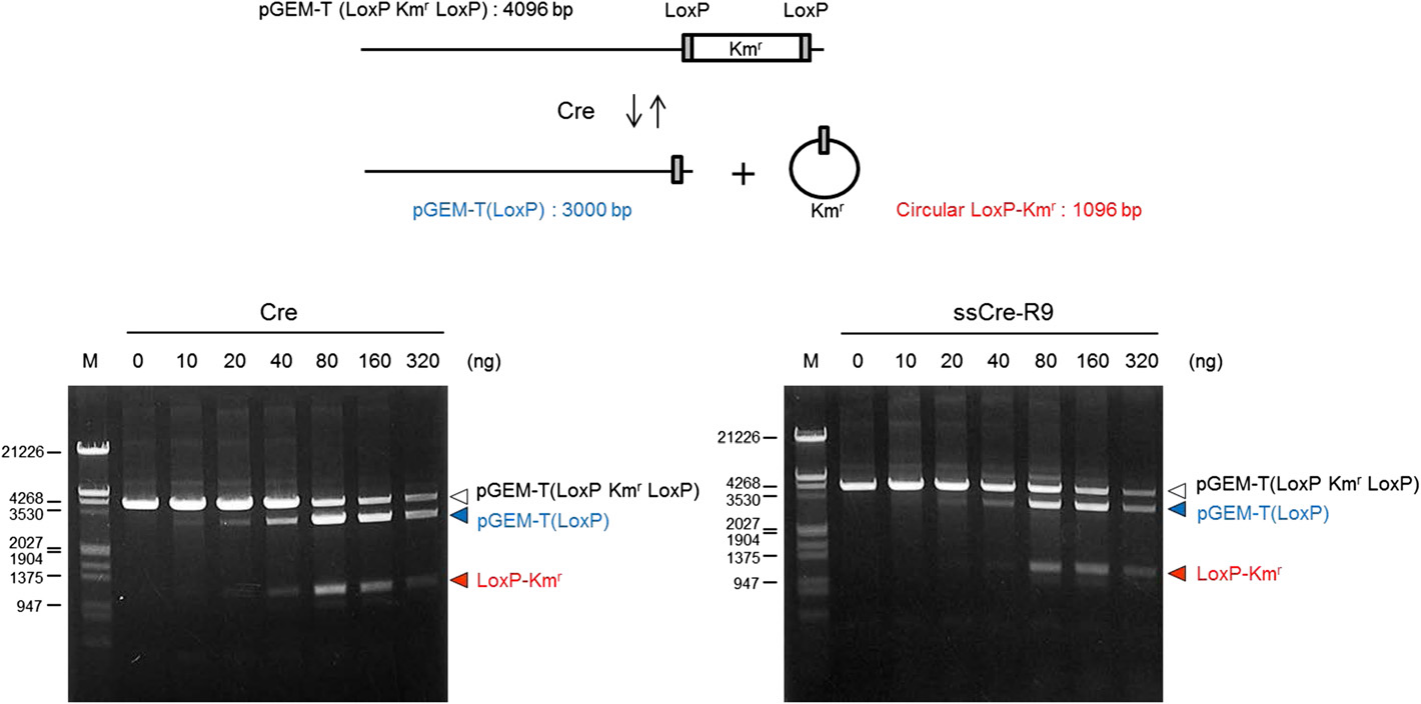
**Activity of Cre-R9 remains intact after EPL.** Linearized pGEM-T (LoxP Km^r^ LoxP) is 4,096 bp in length and contains a LoxP Km^r^ LoxP at one end. Cre-mediated recombination between two LoxP sites results in a linear (3,000 bp) pGEM-T (LoxP) and a circular (1,096 bp) LoxP-Km^r^. Cre and the semisynthetic Cre-R9 (ssCre-R9) were incubated with the linearized pGEM-T (LoxP Km^r^ LoxP) at 37°C for 1 h. After isolated from proteins by phenol extraction, DNA was analyzed by agarose gel electrophoresis. M, Lambda DNA digested with EcoRI and HindIII.

We next determined whether Cre proteins retain their activity after the conjugation of R9 peptides by expressed protein ligation. Control Cre was generated from the Cre-intein-His protein by intein-mediated cleavage with DTT instead of MESNA. Assays of the Cre enzyme activity measured the release of a circular Km^r^ from the linearized pGEM-T with floxed-Km^r^ by the site-specific recombination of Cre (Figure 3). The maximum activity was observed at 80 ng/reaction for both the control Cre and ssCre-R9. This finding demonstrates that the conjugation of CGSGG-R9 to Cre by EPL does not impair the enzymatic activity of Cre.

**Figure 4:**
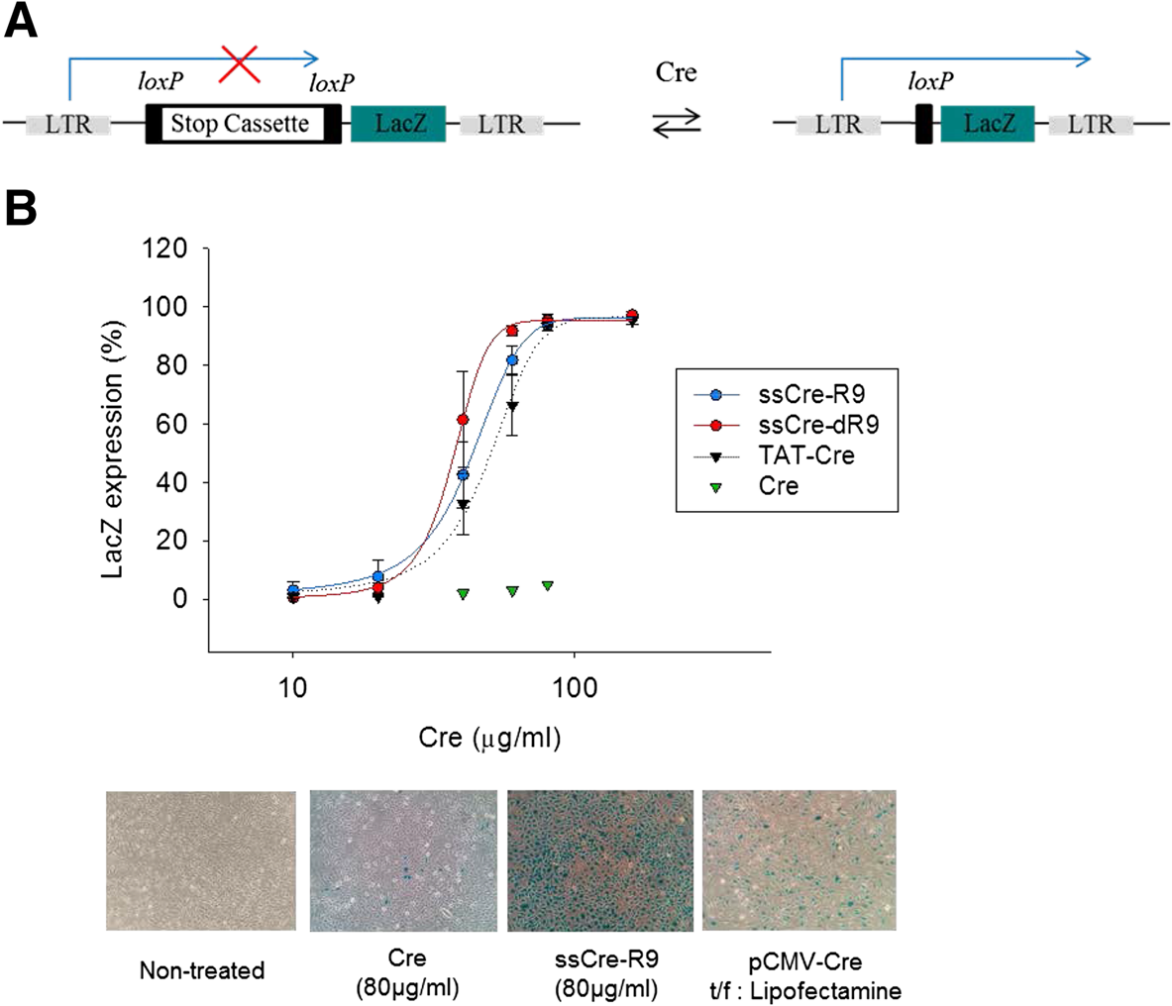
**Protein transduction of the semisynthetic Cre-R9 and Cre-dR9. (A)** Cre-mediated recombination of the floxed STOP cassette leads to LacZ expression in human TE671 (LoxP-LacZ) cells. **(B**) The cells were exposed to varying doses of Cre, ssCre-R9, ssCre-dR9, and TAT-Cre in OPTI-MEM for 5 h. The cells were further incubated with the serum-containing media for a total of 48 h, and then the percentage of LacZ-expressing cells was determined by X-gal staining (n = 4; mean ± SD). The pCMV-Cre expression DNA plasmid was transfected using Lipofectamine.

One major issue in developing a semisynthetic method to produce cell-permeable Cre proteins is whether the functionalities of cell-permeable Cre proteins are maintained during the semisynthetic process. Human TE671 (LoxP-LacZ) cells harbor a loxP-STOP-loxP LacZ reporter gene, which allows us to assess the cellular uptake, subcellular localization and enzymatic activity in the nucleus of Cre proteins (Figure 4A). In order for the transduced Cre proteins to elicit recombination, the exogenous Cre protein is required to enter the cell, translocate to the nucleus, and remove the STOP sequence by recombination at two loxP sites. It results in the expression of *lacZ*, and the cells expressing β-galactosidase turns blue in X-gal staining assay. The transfection of pBS185CMV-Cre plasmid DNA using Lipofectamine induced recombination in 40% of cells (Figure 4). It indicates that Cre proteins without any additional NLS are able to enter the nucleus and elicit recombination in human TE671 cells.

When treated with the control Cre (R9-CPP minus), few *lacZ*-expressing cells were observed (Figure 4). In contrast, treatment with ssCre-R9 showed a dose-dependent increase in recombination. Treatment with 80 μg/ml ssCre-R9 induced recombination in 94% of cells. The EC50_app_ value was measured to be 42.7 μg/ml (1.07 μM) (Table 1). Because 84% of Cre was conjugated to Cre-R9 in the ssCre-R9 preparation (Figure 2B), the EC50_real_ of ssCre-R9 was estimated to be 35.9 μg/ml (0.9 μM). TAT-Cre and Cre-R11 purified as recombinant fusion proteins from *E. coli* are commercially available, and the EC50 of TAT-Cre was measured to be 46.6 μg/ml (1.14 μM) in a parallel experiment (Table 1). Transduction activity of Cre-R11 appeared to be very low; the number of *lacZ*-expressing cells obtained through the treatment of TE671 cells with Cre-R11 was too low to calculate the EC50 value. This result demonstrates that ssCre-R9 is more or at least equally effective for intracellular delivery than the recombinant TAT-Cre or Cre-R11 fusion protein. As reported for the recombinant TAT-Cre protein, the delivery of ssCre-R9 into the cells was also rapid, and treatment with 60 μg/ml ssCre-R9 for 1 h induced recombination in 65% of the cells (Additional file 1: Figure S1). ssCre-R9 was non-toxic to the cells at concentrations up to 160 μg/ml (Additional file 2: Figure S2).

**Table 1:** **EC50 values of Cre recombinase**

**Cre recombinase**	**EC50**_**app**_^***a***^	**EC50**_**real**_^***b***^
ssCre-R9	42.7 ± 3.8 μg/ml (1.07 μM)	35.9 ± 3.2 μg/ml (0.9 μM)
ssCre-dR9	37.2 ± 4.1 μg/ml (0.93 μM)	31.2 ± 3.4 μg/ml (0.78 μM)
TAT-Cre (A company)	-	46.6 ± 6.0 μg/ml (1.14 μM)
Cre-R11 (B company)	-	NA^*c*^

^*a*^Four independent experiments were performed (n = 4; mean ± SD). ^*b*^EC50_real_ is defined as EC50_real_ = EC50_app_ x the conjugation efficiency. ^*c*^NA, not available. The transduction activity of Cre-R11 was too low for EC50_real_ to be measured.

It has been shown that the *d-isomer* R9 peptide labeled with a fluorescent probe had a five-fold lower Km value than the *l-isomer* R9 peptide for cellular uptake [7]. However, the cellular uptake mechanisms of CPPs vary depending on the size and nature of the cargos [24], and it has not been rigorously examined yet whether the *d-isomer* polyarginine CPPs works better than the *l-isomer* polyarginine CPPs for the delivery of large-size protein cargos [20]. In this study, we conjugated CGSGG-dR9 to Cre by EPL and produced ssCre-dR9 with a similar conjugation efficiency, purity, and overall yield as ssCre-R9. To compare the abilities of ssCre-R9 and ssCre-dR9 to enter cells, we treated human TE671 (loxP-LacZ) cells with ssCre-R9 and ssCre-dR9 and scored the recombination events based on the transduced Cre proteins (Figure 4). At concentrations less than 20 μg/ml, ssCre-R9 and ssCre-dR9 were equally non-effective. However, the dose-dependent increase in recombination obtained with ssCre-dR9 was steeper than that obtained with ssCre-R9, and the EC50_app_ values for ssCre-dR9 and ssCre-R9 were 37.2 μg/ml and 42.7 μg/ml, respectively (Table 1). We consistently observed that the EC50 of ssCre-dR9 was at least 10% lower than that of ssCre-R9, although the difference in the EC50 values between ssCre-dR9 and ssCre-R9 was not as marked as the difference in the Km values between the *d-isomer* R9 peptide and the *l-isomer* R9 peptide. Wender et al. measured the initial rates of cellular uptake of the peptides at 3°C for 240 s by FACS after trypsin treatment [7], and this method cannot differentiate the peptides delivered to the cytosol from those incorporated into the plasma membrane or trapped in endosomes. These differences in the assay methods and in the size and nature of the cargos may explain the discrepancy in the extents of the effects of the *d-isomer* on the efficiency of cellular uptake. Nonetheless, these results clearly demonstrate that the *d-isomer* polyarginine is more efficient than the *l-isomer* polyarginine even for the delivery of large-size protein cargos.

**Figure 5:**
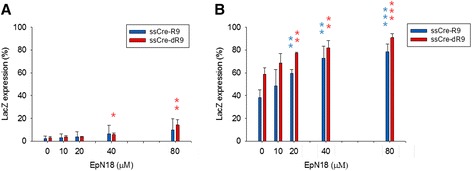
**Enhancement of the transduction of ssCre-R9 and ssCre-dR9 by EpN18.** Human TE671 (LoxP-LacZ) cells were exposed to either 20 μg/m **(A)** or 40 μg/ml **(B)** Cre in the presence of varying amounts of EpN18 for 5 h. The cells were further incubated with the serum-containing media for a total of 48 h, and the expression of LacZ was then examined by X-gal staining (*t*-test; * P < 0.05, ** P < 0.01, *** P < 0.001). Error bars, mean ± SD (n = 3).

The guanidinium group of arginine-rich CPPs forms a bidentate bond with the negative-charged lipid components of a membrane and generates a negative Gaussian membrane curvature, which induces the temporal and dynamic topological transformation of the membrane structure to the *mesh phase* [10,25]. CPPs have been proposed to translocate into the inside surface of a membrane via an adaptive translocation during this phase transition. Therefore, the engineering of the membrane curvature may be a strategy for the promotion of the cellular uptake of CPPs. Indeed, it was found that epsin N-terminal peptide 1–18 (EpN18), a peptide that induces a positive curvature, accelerates the translocation of R8 peptides into cells [26]. However, the effect of membrane curvature on the cellular uptake of CPPs with large-size protein cargos has not yet been examined. In this study, we treated human TE671 (LoxP-LacZ) cells with ssCre-R9 and ssCre-dR9 in the presence of EpN18 and scored the recombination events (Figure 5). EpN18 promotes recombination in a dose-dependent manner when used at a concentration near the EC50 of ssCre-R9 or ssCre-dR9, 40 μg/ml. The acceleration effect of EpN18 started to be observed at 10 μM, and recombination was observed in 91% of cells exposed to 80 μM EpN18 and 40 μg/ml ssCre-dR9. The acceleration effect of EpN18 was also observed in the cells treated with a suboptimal concentration of ssCre-R9 and ssCre-dR9, 20 μg/ml. In this case, however, the acceleration effect of EpN18 became obvious at higher concentrations, such as 40 and 80 μM. Taken together, these results demonstrate that EpN18 is able to promote the cellular uptake of polyarginine CPPs with large-size protein cargos. Because EpN18 is not toxic to cells [26], EpN18 may be useful as a booster for the protein transduction of ssCre-R9 or ssCre-dR9 to cell types that are not amenable to protein transduction.

**Figure 6:**
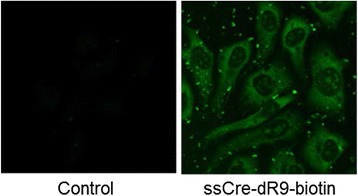
**Cellular imaging of the internalized ssCre-dR9-biotin.** Human TE671 cells were treated with 80 μg/ml ssCre-dR9-biotin for 5 h (ssCre-dR9-biotin) or left untreated (Control). The cells were fixed and then sequentially incubated with avidin-FITC, anti-avidin-biotin antibody, and avidin-FITC. The fluorescence images were taken under a confocal laser scanning microscope.

The visualization of the cells transduced with therapeutic cell-permeable proteins will be highly advantageous for post-treatment evaluation. As the first step to explore this possibility, we synthesized dR9-biotin [NH2-Cys-Gly-Ser-Gly-Gly-D (Arg)9-Gly-Gly-Lys (biotin)-amide] and conjugated dR9-biotin to Cre by EPL. ssCre-dR9-biotin was produced with a similar purity and overall yield as ssCre-dR9. We found little difference in the transduction efficiency between ssCre-dR9 and ssCre-dR9-biotin. To visualize the ssCre-dR9-biotin delivered into cells, the cells were treated with ssCre-dR9-biotin for 5 h, washed with PBS, fixed with paraformaldehyde, and then stained with avidin-FITC. Images taken using a confocal laser scanning microscope showed both cytoplasmic and nuclear staining with a distinct pattern (Figure 6). A previous study raised the possibility that cell fixation leads to the artifactual redistribution of CPP [27]. Therefore, it is unlikely that ssCre-dR9-biotin has the same subcellular localization in living cells demonstrated by avidin-FITC staining in this study. Nonetheless, these results clearly demonstrated the applicability of EPL for the preparation of cell-permeable proteins with biotin for cell imaging. For *in vivo* imaging of whole animals or cells, it will be more advantageous to use CPPs with fluorophores instead of biotin. It will be interesting to devise a near-infrared (NIR) fluorophore that can be located near CPP without hampering the delivery and activity of proteins in cells.

## Conclusions

In this study, we devised an EPL-based technique to conjugate synthetic polyarginine CPPs to Cre protein expressed in *E. coli* without impairing the enzymatic activity of Cre. The ligation product, ssCre-R9, exhibited comparable or even better transduction activity compared with the recombinant TAT-Cre and Cre-R11 fusion proteins directly expressed in *E. coli*. Because all of the steps of EPL are designed to proceed in the presence of high salt concentrations, this approach avoids the technical hurdle caused by the decrease in the solubility of polyarginine fusion proteins when expressed in *E. coli*. In addition, this EPL-based technique allowed us to prepare ssCre-dR9 and ssCre-dR9-biotin containing poly-*D*-arginine and biotin as part of the CPPs. ssCre-dR9 resulted in improved protein transduction compared with Cre-R9, and ssCre-dR9-biotin allowed the visualization of the cells transduced with ssCre-dR9-biotin. The findings demonstrated the feasibility of this EPL-based technique for the preparation of cell-permeable proteins with higher transduction efficiency and more elaborate properties for use in more sophisticated applications, such as cell imaging. EPL should find broad use for the introduction of cell permeability to therapeutic proteins, such as transcription factors or antibodies.

## Methods

### Peptides

CGSGG-R9 [NH2-Cys-Gly-Ser-Gly-Gly-(Arg) 9-amide], CGSGG-dR9 [NH2-Cys-Gly-Ser-Gly-Gly-D (Arg) 9-amide], EpN18 [26], and dR9-biotin [NH2-Cys-Gly-Ser-Gly-Gly-D (Arg) 9-Gly-Gly-Lys (biotin)-amide] were synthesized and provided by Peptron Inc. The peptides were purified by high-performance liquid chromatography to a purity >90% and confirmed by mass analysis.

### Preparation of the Cre-intein fusion protein

Cre recombinase DNA was obtained from pBS185CMV-Cre (Addgene plasmid 11916) through two amplification steps, first using the forward primer 5′-GAG CAT ATG TCC AAT TTA CTG ACC GTA CAC-3′ and the reverse primer 5′-GTA CTC GAG ATC GCC ATC TTC CAG GAC-3′ and then using the forward primer 5′-GAG CAT ATG TCC AAT TTA CTG ACC GTA CAC-3′ and the reverse primer 5′-GCG TGC TCT TCC GCA GTA CTC GAG ATC GCC ATC-3′ [28]. The amplified DNA was inserted into the Nde I and Sap I sites on the pTWIN_His vector (Jena Bioscience) to construct an expression plasmid for a recombinant fusion protein of Cre and mini-intein (derived from Mycobacterium Xenopi gyrase A, Mxe GyrA) combined with a 6× His-tag. Leu-Glu-Tyr was inserted between Cre and intein to improve the intein-mediated cleavage. The pTWIN_Cre plasmid encoding the Cre-intein-His fusion protein was transformed into *E. coli* BL21 (DE3) cells. Then, 50-ml LB medium overnight culture were inoculated into 1 L of LB medium with ampicillin (50 μg/ml) and grown at 37°C. When OD_600nm_ reached to 0.6, yeast extract was added to 1% and then the expression of Cre-intein-His was induced by 1 mM isopropyl-β-D-thiogalactopyranoside (IPTG) at 37°C for 3 h. The *E. coli* cells were harvested by centrifugation and resuspended with 5× packed cell volume of lysis buffer (20 mM NaH_2_PO_4_, 300 mM NaCl, and 10 mM imidazole, pH 8.0), treated with 1 mg/ml lysozyme on ice for 30 min, and then lysed via sonication. The soluble supernatant was collected by centrifugation at 10,000 × g and mixed with 10 ml of the 50% slurry of Ni-NTA resin (Qiagen) at 4°C for 1 h. The resin slurry was packed into a column and washed with five column volumes of washing buffer (50 mM NaH_2_PO_4_, 300 mM NaCl, and 50 mM imidazole, pH 8.0). The Cre-intein-His protein was eluted from the column with elution buffer (50 mM NaH_2_PO_4_, 300 mM NaCl, and 250 mM imidazole, pH 8.0). The fractions containing the Cre-intein-His protein were combined and dialyzed against cleavage buffer (50 mM NaH_2_PO_4_ and 250 mM NaCl, pH 8.0) at 4°C overnight.

### Intein-mediated cleavage and ligation of Cre-thioester with polyarginine polypeptides

The intein-mediated cleavage to generate the Cre-thioester and the ligation of the Cre-thioester with polyarginine polypeptides were performed at the same time. The Cre-intein-His protein in cleavage buffer (50 mM NaH_2_PO_4_ and 250 mM NaCl, pH 8.0) was incubated with 50 mM 2-mercaptoethanesulfonate (MESNA) and a 10-fold molar excess of CGSGG-R9 or CGSGG-dR9 at 4°C for 24 h. To separate Cre-R9 from the Cre-intein-His or intein-His proteins, the reaction mixture was loaded onto a Ni-NTA column equilibrated with lysis buffer, and the flow-through fraction was collected. Cre-R9 was treated with dithiothreitol (DTT) to a final concentration of 10 mM, incubated on ice for 30 min, and then concentrated using a Pierce concentrator 9K (Thermo) at 4°C. Any leftover MESNA and peptides in Cre-R9 were removed by a spin column packed with Sephadex G25 Fine resin GE Healthcare) equilibrated with storage buffer (50 mM NaH_2_PO_4_, 300 mM NaCl, and 1 mM DTT, pH 8.0).

### Cell culture

Human TE671 (LoxP-LacZ) cells were purchased from Allele Biotech. The cells were cultured at 37°C in a 5% humidified CO_2_ incubator in Dulbecco’s Modified Eagle’s medium (DMEM) with 10% fetal bovine serum, 100 U/ml penicillin G, and 100 μg/ml streptomycin.

### Protein transduction assay

TAT-Cre and Cre-R11 were purchased commercially. To measure the rate of Cre-R9 internalization and subsequent recombination, human TE671 (LoxP-LacZ) cells were plated onto 24-well plate at 7 × 10^3^ cells/well. After one day, the cells were washed once with OPTI-MEM (Gibco) and incubated with 300 μl of OPTI-MEM containing Cre proteins for 5 h. The medium was changed to DMEM containing 10% FBS, and the cells were then further incubated for 43 h. The recombination at LoxP sites by Cre proteins in the cells was measured by a LacZ enzyme assay. The cells were washed twice with cold PBS and fixed with 300 μl of a fixation solution (1% formaldehyde and 0.2% glutaraldehyde in PBS) at room temperature for 5 min. The cells were washed three times with PBS and incubated with 300 μl of an X-gal staining solution (4 mM K-ferrocyanide, 4 mM K-ferricyanide, 2 mM MgCl_2_, and 0.4 mg/ml X-gal in PBS) at 37°C overnight.

### *In vitro* assay of Cre activity

Cre protein was added to 50 μl of reaction buffer (50 mM Tris–HCl, 33 mM NaCl, and 10 mM MgCl_2_, pH 7.5) containing 0.3 μg of linearized pGEM-T (floxed-Km^r^) and incubated at 37°C for 1 h. Cre was then heat-inactivated by incubation at 70°C for 10 min. After isolation from proteins by phenol extraction and EtOH precipitation, DNA was analyzed by agarose gel electrophoresis.

### Fluorescence imaging

Human TE671 cells grown on gelatin-coated glass coverslips were incubated with 80 μg/ml ssCre-dR9-biotin in OPTI-MEM for 5 h. The cells were washed three times with PBS and fixed in 3.7% (v/v) paraformaldehyde for 15 min at room temperature. The cells were sequentially incubated with avidin-FITC, anti-avidin-biotin antibody, and avidin-FITC each for 30 min at room temperature. After mounting with 90% glycerol and 0.1% *o*-phenylenediamine in PBS, cellular images were obtained using a confocal laser scanning microscope.

## Additional files

Additional file 1: Figure S1. One hour of incubation with exogenous ssCre-R9 is sufficient for Cre-mediated recombination in human TE671 (LoxP-LacZ) cells. Cells were exposed to 60 μg/ml of ssCre-R9 in OPTI-MEM for the indicated time. Cells were further incubated with the serum-containing media for the total 48 h, and then the expression of LacZ was examined by X-gal staining (n = 4; mean ± SD). Additional file 2: Figure S2. ssCre-R9 does not affect cell viability. Human TE671 (LoxP-LacZ) cells were incubated with ssCre-R9 in OPTI-MEM for 5 h and further incubated with the serum-containing media for a total of 48 h. Cell viability was measured by MTT assay (n = 3; mean ± SD).

## Abbreviations

R12: Arginine 12-mer
CPP: Cell-penetrating peptide
EGFP: Enhanced green fluorescent protein
EPL: Expressed protein ligation
EpN18: Epsin N-terminal peptide 1–18
NLS: The nuclear localization sequence
ssCre-R9: Semisynthetic Cre-R9
