# Supplementary figures and images for: Preparation of cell-permeable Cre recombinase by expressed protein ligation
Source: BMC Biotechnol. 2015 Feb 19;15(1):7. doi: 10.1186/s12896-015-0126-z (PMC4339299; doi:10.1186/s12896-015-0126-z)

## Slide 1
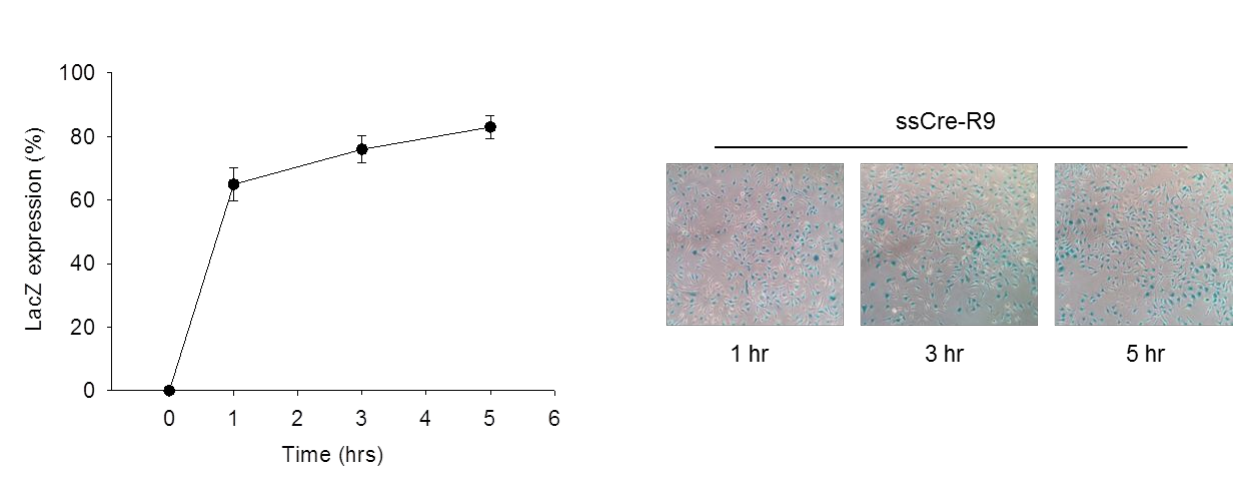

Supplement: Additional file 1: Figure S1. — One hour of incubation with exogenous ssCre-R9 is sufficient for Cre-mediated recombination in human TE671 (LoxP-LacZ) cells. Cells were exposed to 60 μg/ml of ssCre-R9 in OPTI-MEM for the indicated time. Cells were further incubated with the serum-containing media for the total 48 h, and then the expression of LacZ was examined by X-gal staining (n = 4; mean ± SD). [file 12896_2015_126_MOESM1_ESM.pptx]

## Slide 1
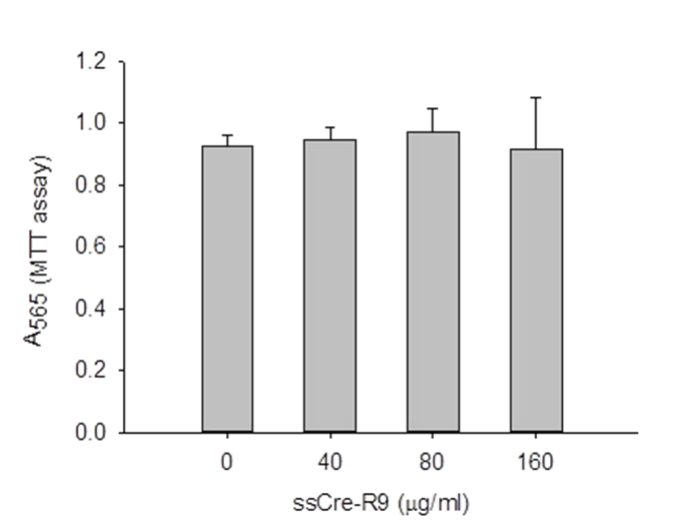

Supplement: Additional file 2: Figure S2. — ssCre-R9 does not affect cell viability. Human TE671 (LoxP-LacZ) cells were incubated with ssCre-R9 in OPTI-MEM for 5 h and further incubated with the serum-containing media for a total of 48 h. Cell viability was measured by MTT assay (n = 3; mean ± SD). [file 12896_2015_126_MOESM2_ESM.pptx]
